# Efficient generation of single-copy transgenic mice using *piggyBat* transposase from the little brown bat *Myotis lucifugus*^†^

**DOI:** 10.1093/biolre/ioaf235

**Published:** 2025-09-13

**Authors:** Eiichi Okamura, Shoma Matsumoto, Hayate Suzuki, Yoko Tanimoto, Tra Thi Huong Dinh, Natsuki Mikami, Masanaga Muto, Tomoko Matsumoto, Fumihiro Sugiyama, Satoru Takahashi, Knut Woltjen, Seiya Mizuno, Masatsugu Ema

**Affiliations:** Department of Stem Cells and Human Disease Models, Research Center for Animal Life Science, Shiga University of Medical Science, Otsu, Shiga, Japan; Department of Stem Cells and Human Disease Models, Research Center for Animal Life Science, Shiga University of Medical Science, Otsu, Shiga, Japan; Laboratory Animal Resource Center in Trans-border Medical Research Center, University of Tsukuba, Tsukuba, Ibaraki, Japan; Laboratory Animal Resource Center in Trans-border Medical Research Center, University of Tsukuba, Tsukuba, Ibaraki, Japan; Laboratory Animal Resource Center in Trans-border Medical Research Center, University of Tsukuba, Tsukuba, Ibaraki, Japan; Laboratory Animal Resource Center in Trans-border Medical Research Center, University of Tsukuba, Tsukuba, Ibaraki, Japan; Ph.D. Program in Human Biology, School of Integrative and Global Majors, University of Tsukuba, Tsukuba, Ibaraki, Japan; Japan Society for the Promotion of Science, Tokyo, Japan; Department of Stem Cells and Human Disease Models, Research Center for Animal Life Science, Shiga University of Medical Science, Otsu, Shiga, Japan; Department of Life Science Frontiers, Center for iPS Cell Research and Application (CiRA), Kyoto University, Kyoto, Japan; Laboratory Animal Resource Center in Trans-border Medical Research Center, University of Tsukuba, Tsukuba, Ibaraki, Japan; Laboratory Animal Resource Center in Trans-border Medical Research Center, University of Tsukuba, Tsukuba, Ibaraki, Japan; Department of Anatomy and Embryology, Institute of Medicine, University of Tsukuba, Tsukuba, Ibaraki, Japan; International Institute for Integrative Sleep Medicine (IIIS), Life Science Center (TARA), University of Tsukuba, Tsukuba, Ibaraki, Japan; Department of Life Science Frontiers, Center for iPS Cell Research and Application (CiRA), Kyoto University, Kyoto, Japan; Laboratory Animal Resource Center in Trans-border Medical Research Center, University of Tsukuba, Tsukuba, Ibaraki, Japan; Department of Stem Cells and Human Disease Models, Research Center for Animal Life Science, Shiga University of Medical Science, Otsu, Shiga, Japan; Institute for the Advanced Study of Human Biology (WPI-ASHBi), Kyoto University, Kyoto, Japan

**Keywords:** pronuclear injection, transgenic animal, mouse, *piggyBat*, *piggyBac*, zygote

## Abstract

Transgenic animals are invaluable tools in genetic studies, disease modeling, drug discovery, and biotechnology. However, the low efficiency of transgenic animal generation can be an obstacle to their application. Here, we report the generation of transgenic mice using PBatase, the *piggyBat* transposase from the little brown bat (*Myotis lucifugus*). PBatase exhibits detectable transposition activity in fertilized mouse eggs within a limited concentration range, although the overall activity was lower than that of PBase, the *piggyBac* transposase from the cabbage looper moth (*Trichoplusia ni*). Transgenic animals carrying low transgene copy numbers were successfully generated with high efficiency using PBatase, and the transgene was subsequently transmitted to the next generation. This technique will be useful for the generation of transgenic animals carrying single copies of a transgene.

## Teaser

Efficient generation of transgenic animals with single-copy transgenes using *piggyBat*, a transposase from the little brown bat, *Myotis lucifugus*.

## Graphical Abstract

**Graphical Abstract:**
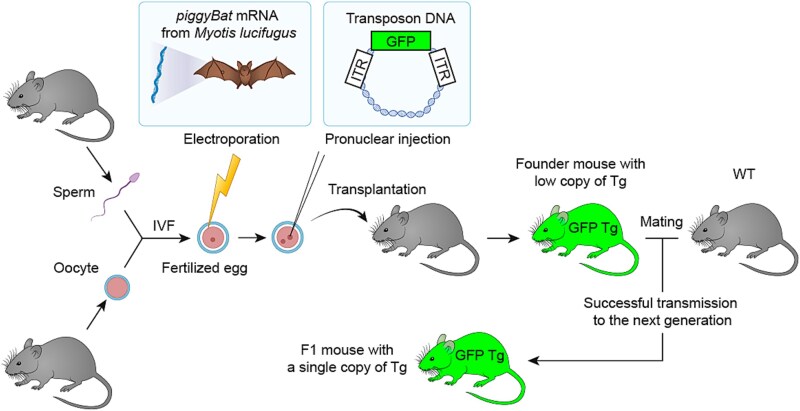

## Introduction

The generation of transgenic animals, including mice, rats, and other domestic animals, has contributed greatly to understanding the pathogenesis and molecular mechanisms of human disease [1–3]. The first transgenic mouse was generated by retroviral vector infection [4]. Subsequently, transgenic animals were generated by pronuclear injection of double-stranded DNA into fertilized eggs [5, 6], and this is still the most popular strategy used today. This technique, however, has the disadvantages of low efficiency (20% at best) [7] and the insertion of variable numbers of transgene copies because of transgene concatemer formation by DNA ligation [8].

*piggyBac* is a DNA transposon system that was originally discovered in the genome of the cabbage looper moth (*Trichoplusia ni*) and has become a powerful tool for genetic engineering [9]. It can mobilize a segment of DNA from one location to another within a genome, by “cutting” the transposon out of its original location and “pasting” it into a new location within the genome [9]. The *piggyBac* transposase enzyme (PBase) recognizes specific short sequences called inverted terminal repeats (ITRs) to excise the transposon from its original location [10]. The *piggyBac* system is widely used to introduce multiple copies of a gene of interest into various animal models with high efficiency, in some cases achieving 100% transgenesis [11]. However, the introduction of multiple transgene copies throughout the genome may be disadvantageous for the establishment of stable transgenic lines with a single transgene insertion site due to random segregation during conventional breeding. Moreover, transgenes carrying a gene of interest flanked by recombinase sites such as loxP should be introduced into the genome in a single copy to avoid the potential for inter- or intra-chromosomal recombination.

*piggyBat* is a recently discovered transposable element found in the genome of the little brown bat (*Myotis lucifugus*) [12]. It is a member of the *piggyBac* superfamily and has similar properties to *piggyBac* transposons in that it can cut and paste itself into different parts of the genome, with a high specificity (>90%) for TTAA tetranucleotides [12]. *piggyBat* has been used to introduce chimeric antigen receptors into human T cells for potential gene therapeutic purposes [13]. However, unlike *piggyBac*, *piggyBat* has not been well characterized or used to generate transgenic animals.

Here, we report the transposition of *piggyBat* in mouse embryonic stem cells (ESCs) and the direct generation of transgenic mice using *piggyBat* into fertilized mouse eggs. *piggyBat* transposition activity is generally moderate, and the number of transgene copies inserted into a genome is usually one or two, enabling the generation of transgenic animals with a single copy of the transgene. Subsequently, the inserted *piggyBat* transgene was successfully transmitted to the next generation. Therefore, *piggyBat* is useful for generating transgenic animals carrying a single copy of the transgene.

## Materials and methods

### Plasmids

pCAG-mPBase (KW158) is described previously [14]. PB53-CMV early enhancer/chicken beta actin (CAG)-Green Fluorescent Protein (GFP)-puro (KW148) and pCAG-PBatase (KW398) will be described elsewhere. The CAG promoter-GFP-IRES-puroR cassette flanked by the mPBase and pPBatase ITRs was constructed by inserting the CAG promoter-GFP-IRES-puroR cassette from KW148 into an in vitro synthesized DNA fragment containing mPBase and pPBatase ITRs (Eurofin Inc., Tokyo, Japan). PBSplitGFP, in which GFP is split by inverted mPBase ITRs [15], was a gift from Dr. Robi David Mitra (Washington University School of Medicine, St Louis, USA). A plasmid overexpressing H2B-GFP-P2A-membranous mCherry under the control of the CAG promoter was constructed by inserting CAG promoter-H2B-GFP-P2A-membranous mCherry between pPBatase ITRs. A 5.5 kb myosin heavy-chain promoter fragment (a kind gift from W. L. Stanford, The Ottawa Hospital Research Institute, Ottawa, Canada) was ligated to GFP-pA and further ligated in the opposite orientation to the CAG promoter-NLS-tdTomato cassette. cHS4, a core insulator sequence, was then inserted into the flanking region [16].

For the synthesis of mPBase and PBatase mRNAs, the Open Reading Frame (ORF) sequences of mPBase and PBatase were cloned into the pmRNA plasmid vector (TriLink BioTechnologies (TriLink BioTechnologies LLC, San Diego, CA, USA), which contains a synthetic 5′ untranslated region (UTR) and a mouse Hba-a1 gene 3′ UTR, as described in Supplementary Figure 1. The mRNAs were synthesized in vitro using T7 RNA polymerase–mediated transcription by TriLink BioTechnologies (TriLink BioTechnologies LLC).

### Mouse embryonic stem cell culture and transposition assay

Mouse ESCs carrying the zeocin resistance gene in the *Oct3/4* locus [17] were used to maintain pluripotent stem cell populations in ESC medium [DMEM (Thermo Fisher Scientific, Waltham, MA, USA, 10565-018) supplemented with 10% fetal bovine serum, 1 mM sodium pyruvate, 0.1 mM 2-mercaptoethanol (Sigma-Aldrich M3148), 1× non-essential amino acids (Thermo Fisher Scientific, 11140-050), 1 mM L-glutamine, 100 U/ml penicillin/streptomycin (Thermo Fisher Scientific, 15140-122), and 1000 U leukemia inhibitory factor/ml] in the presence of zeocin (20 ng/ml).

For the transposition assay, a total of 2 μg of DNA (0.5 μg of the transposon DNA and various doses of the transposase DNA; mPBase for *piggyBac* and PBatase for *piggyBat*) was introduced into 0.4 million cells of mESCs in a 24 well by using Lipofectamine 2000 (Thermo Fisher Scientific). Twenty-four hours after the transfection, the cells were plated on a 6-well plate and cultured in the presence of 2 μg/ml puromycin for 5 days, and the numbers of resistant colonies were counted.

For the excision assay, a total of 2 μg DNA (0.5 μg of the reporter DNA and various doses of the mPBase PBatase) was introduced into 0.4 million cells of mESCs and cultured for 2 days, followed by flow cytometric analysis using a FACSCalibur cytometer (BD Biosciences).

### Animal experiments

All experimental procedures were approved by the Animal Care and Use Committee of Shiga University of Medical Science (approval number: 2020-6-21) and the Institutional Animal Experiment Committee of Tsukuba University (approval number: 22-019).

C57BL/6 J and ICR mice were obtained from Charles River Laboratories (Yokohama, Japan). Mice were housed in plastic cages under pathogen-free conditions in a room maintained at 23.5 ± 2.5°C and 52.5 ± 12.5% relative humidity with a 14:10-hour light:dark cycle.

Fertilized eggs were obtained by in vitro fertilization using a method described elsewhere [11]. Briefly, oocytes were collected from oviducts of 10–14-week-old C57BL/6 J females superovulated by the intraperitoneal administration of CARD HyperOva (Kyudo, Tosu, Japan), followed by human chorionic gonadotropin. Sperm were collected from the caudal epididymis of 12–16-week-old C57BL/6 J males and then preincubated in Fertiup Mouse Sperm Preincubation Medium (Kyudo). Fertilization was performed in CARD medium (Kyudo), followed by incubation at 37°C in an atmosphere containing 5% CO_2_ for 3–6 h. Fertilized eggs were washed to remove cumulus cells and sperm and incubated in K+ Simplex Optimised Medium (KSOM) medium (Ark Resource, Kumamoto, Japan) until electroporation.

Electroporation of fertilized eggs was performed in Opti-MEM I medium (Thermo Fisher Scientific) containing *piggyBat* or *piggyBac* mRNA using a Super Electroporator NEPA21 (Nepa Gene, Ichikawa, Japan) as described elsewhere [11] with minor modifications. In this study, the poring pulse was set as: 225 V, 10% attenuation rate, 2-ms pulse width, 50-ms pulse interval, 4 pulses, and + polarity. The transfer pulse was set as: 20 V, 50-ms pulse width, 50-ms pulse interval, 5 pulses, 40% attenuation rate, and ± polarity. The eggs were incubated until pronuclei become clearly visible. The transposon DNA was injected into male pronuclei under a microscope equipped with a micromanipulator and a Femtojet (Eppendorf, Hamburg, Germany). The injected one-cell embryos were cultured in KSOM medium until the two-cell stage and then transferred to pseudopregnant ICR females or cultured to the blastocyst stage.

The GFP fluorescence intensity of mouse blastocysts was analyzed using Fiji software by defining a region of interest and subtracting the signal from that of a wild-type blastocyst–injected transposon plasmid without PBatase messenger RNA (mRNA) introduction. Embryos with a positive value were considered GFP-positive.

### Genotyping

Embryonic day (E)9.5 and E11.5 embryos were dissected from the uteri of euthanized surrogate mothers. The placenta and yolk sac were removed from each fetus. Fluorescence signals in tissues were captured under a fluorescence stereoscopic microscope (M165FC; Leica Microsystems, Wetzlar, Germany) equipped with a camera (VB-7010; Keyence, Osaka, Japan). Genomic DNA was extracted from the embryos by phenol/chloroform extraction. Genotyping was performed by conventional Polymerase Chain Reaction (PCR) using a T100 thermal cycler (Bio-Rad, Hercules, CA, USA) and a primer set (GFP q656Rn1: 5′-acgtaaacggccacaagttc-3′, GFP q470Fn1: 5′-aagtcgtgctgcttcatgtg-3′, 187 bp fragment) to detect the GFP sequence. Primer sets for *Klf5* (Klf5 5′-2: 5′-aggcctgataaaataacctagtcca-3′, Klf5 WT allele 3: 5′-cgacatgtcttcactaaagtcactc-3′, 302 bp fragment) or *Rbm31* (Rbm31-Sexing-F: 5′-caccttaagaacaagccaataca-3′, Rmb31-Sexing-R: 5′-ggcttgtcctgaaaacatttgg-3′, 269 bp fragment for the X chromosome, 353 bp fragment for the Y chromosome) were used as positive controls for mouse genomic DNA.

To determine the transgene copy number, droplet digital PCR (ddPCR) was performed using the QX200 Droplet Digital PCR System (Bio-Rad) with ddPCR Supermix for probes (No dUTP) (Bio-Rad) according to the manufacturer’s instructions. The PrimeTime qPCR probe with 5′ FAM and 3′ IBFQ quencher, in combination with the internal ZEN quencher and primers to detect the GFP sequence, was synthesized by Integrated DNA Technologies (IDT, Tokyo, Japan) as follows: GFP probe FAM: 5′-ACGACGGCAACTACA-3′, GFP primer 1: 5′-GAGCGCACCATCTTCTTCAAG-3′, GFP primer 2: 5′-TGTCGCCCTCGAACTTCAC-3′. TaqMan Copy Number Reference Assay, mouse, Tfrc (Thermo Fisher Scientific Cat#4458366) was used as an internal control.

### Southern blotting

Genomic DNA was extracted from mouse embryos with phenol and chloroform and then purified by ethanol precipitation. After digestion of 5 μg of genomic DNA with a restriction enzyme, the DNA concentration was measured using a Quantus Fluorometer (Promega, Madison, WI, USA). Digested DNA was electrophoresed on a 1% agarose gel. The DNA was blotted onto a nylon membrane (Amersham Hybond-N^+^; GE Healthcare, Chicago, IL, USA) and hybridized with Digoxigenin (DIG)-labeled probes using DIG Easy Hyb (Roche Diagnostics, Mannheim, Germany). The DIG-labeled probes were synthesized by PCR using DIG DNA Labeling Mix, 10× Conc. (Roche). Salmon sperm DNA was used in the blocking step. After reaction with anti-digoxigenin-AP Fab fragments (Roche), bands were detected by enhanced chemiluminescence using CDP-Star (Roche) and ImageQuant LAS 4000 Mini (GE Healthcare).

### Statistical analysis

Statistical analysis of data comparisons was performed by the Tukey–Kramer test and Brown–Forsythe test using R packages (https://www.r-project.org/). *P* < 0.05 was considered statistically significant.

## Results

### Evaluation of piggyBat transposition activity in mouse embryonic stem cells

**Figure 1:**
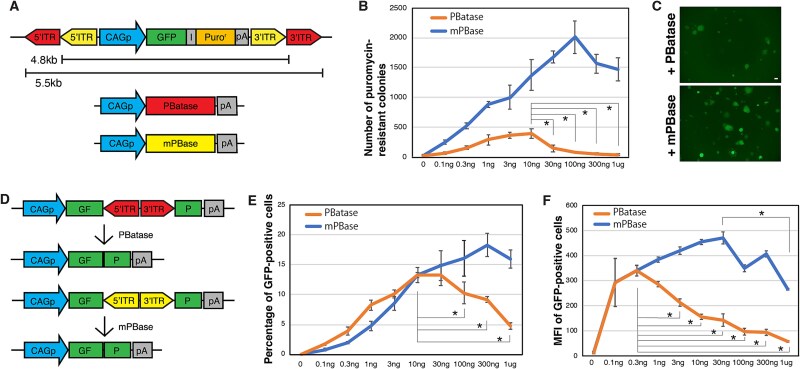
Dose-dependent transposition activity of PBatase. (A) Schematic representation of the transposon and transposase vectors for the transposition assay. GFP is driven by the CAG promoter. An IRES (I) and puromycin resistance gene are inserted downstream of GFP. The ITRs for *piggyBac* and *piggyBat* are shown. (B) Transposition activities of *piggyBac* and *piggyBat* measured by the number of puromycin-resistant colonies. (C) Representative images for GFP-positive mESC colonies generated by *piggyBac* and *piggyBat*. Scale bars = 100 μm. (D) Schematic of the assay to measure the excision activity of the transposases. (E) Percentage of GFP-positive cells in the excision assay as measured by flow cytometry. (F) Mean fluorescent intensity (MFI) of GFP-positive cells in the excision assay. The mean values (±SD) of biological triplicates. ^*^, *P* < 0.05.

To achieve our goal of developing a technique to generate transgenic animals using *piggyBat*, we first aimed to evaluate the transposition activity of *piggyBat* in vitro. A *piggyBat* transposon DNA carrying GFP and a puromycin-resistance gene driven by a ubiquitous CAG promoter was introduced into mouse ESCs together with various doses (0–1 μg) of PBatase expression plasmid DNA and cultured in the presence of puromycin for 5 days (Figure 1A). *piggyBac* transposition with a mouse codon-optimized PBase (mPBase) was used as a control because it has been extensively characterized and used to generate transgenic cultured cells and animals [9]. Both *piggyBac* and *piggyBat* produced a significant number of drug-resistant colonies (Figure 1B and C). In contrast to *piggyBac*, in which the activity peaks at 30–300 ng, *piggyBat* activity peaked at approximately 3 and 10 ng, and then decreased to less than 10% of the peak activity with increasing dose up to 1 μg (Figure 1B). Overall, *piggyBat* exhibited lower transposition activity than *piggyBac* across most tested concentrations but maintained detectable activity within a moderate-dose window (approximately 0.3–10 ng), before being decreased at higher levels.

Estimation of transposition activity from drug-resistant colonies is a commonly used approach. However, puromycin-resistant colonies are generated by at least one integrated transposon [15]. Therefore, we employed an alternative method to evaluate transposition activity in which a donor vector in which GFP is interrupted and inactivated by a transposon (Figure 1D) [15]. The transposase excises the transposon, creating a functional GFP, and therefore, the percentage of GFP-positive cells is proportional to the number of transposon excision events [15]. *piggyBat* up to 10 ng increased the percentage of GFP-positive cells, which then decreased with increasing dose, suggesting overproduction inhibition (OPI), whereas *piggyBac* increased the percentage of GFP-positive cells with increasing dose (Figure 1E). *piggyBat* up to 0.3 ng increased the mean fluorescent intensity (MFI) of GFP-positive cells, which then decreased with increasing dose, suggesting OPI, whereas *piggyBac* also showed slight OPI around 1 μ g (Figure 1F). This is consistent with the results of the transposition assay assessing drug-resistant colonies (Figure 1B). The transposon excision capacity of *piggyBat* is similar to that of *piggyBac* at low doses (0.1–10 ng) and lower at high doses (100 ng–1 μg) (Figure 1E).

### Generation of transgenic mice with piggyBat

**Figure 2:**
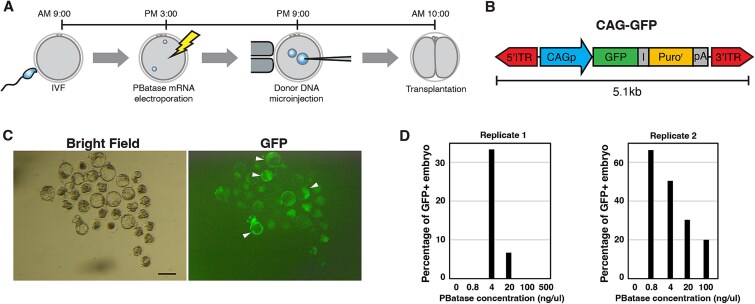
Optimization of PBatase transposition activity in fertilized mouse eggs. (A) Schedule for pronuclear injection of the transposon DNA. (B) Schematic representation of the transposon, CAG-EGFP-IRES-Puro (CAG-GFP) donor DNA, used to generate transgenic embryos. I; IRES, ITR: ITR for PBatase. (C) Detection of GFP fluorescence in mouse blastocysts developed in vitro. White arrowheads show GFP-positive blastocysts, which were defined by the intensity of GFP measured using ImageJ. (D) Percentage of GFP-positive blastocysts after introduction of the CAG-GFP donor DNA together with different doses of PBatase. Two independent biological replicates were performed and are plotted separately. Scale bars = 100 μm.

The differential transposition activity of PBatase doses in cultured cells prompted us to investigate the optimal PBatase dose for transposition activity in fertilized mouse eggs by introducing different doses of the transposase mRNA. We electroporated fertilized eggs with PBatase mRNA 6 h before the pronuclear injection of transgene DNA, so that the PBatase could express and act on the transgene DNA immediately as it was introduced into the nucleus (Figure 2A). Introduction of various doses of PBatase (0, 0.8, 4, 20, 100, and 500 ng/ul) together with a *piggyBat* transposon–carrying GFP driven by the ubiquitous CAG promoter (Figure 2B) showed peak activity at 4 ng/μl in replicate 1 (Figure 2C and D, Table 1), consistent with the in vitro activity (Figure 1). In replicate 2, 0.8 ng/μl yielded the highest activity, whereas 4 ng/μl consistently exhibited the second-highest activity (Figure 2D, Table 1). Because 4 ng/μl reproducibly achieved high efficiency across both replicates, we regarded it as the optimal PBatase dose in preimplantation embryos.

**Table 1:** Summary of piggyBat mRNA concentration optimization

**Experiment**	**mRNA conc. (ng/μl)**	**Number**						
		**Electroporated**	**Survived (% of electroporated)**	**Microinjected**	**Survived (% of microinjected)**	**2-cell stage (% of survived)**	**Blastocyst (% of survived)**	**Transgenic (% of blastocyst)**
1st	0	35	31 (88.6)	31	27 (87.1)	NA	16 (59.3)	0 (0.0)
	0.8	35	33 (94.3)	33	29 (87.9)	NA	4 (13.8)	0 (0.0)
	4	35	33 (94.3)	33	32 (97.0)	NA	12 (37.5)	4 (33.0)
	20	35	34 (97.1)	34	30 (88.2)	NA	15 (50.0)	1 (66.7)
	100	35	33 (94.3)	33	32 (97.0)	NA	21 (65.6)	0 (0.0)
	500	35	32 (91.4)	32	21 (65.6)	NA	8 (38.1)	0 (0.0)
2nd	0	40	39 (97.5)	39	36 (92.3)	35	22 (61.1)	0 (0.0)
	0.8	40	40 (100)	40	36 (90.0)	36	25 (69.4)	16 (64.0)
	4	40	40 (100)	40	36 (90.0)	32	22 (61.1)	11 (50.0)
	20	40	40 (100)	40	37 (92.5)	35	19 (51.4)	6 (31.6)
	100	40	37 (92.5)	37	34 (91.9)	27	15 (44.1)	3 (20.0)

NA: not available

**Figure 3:**
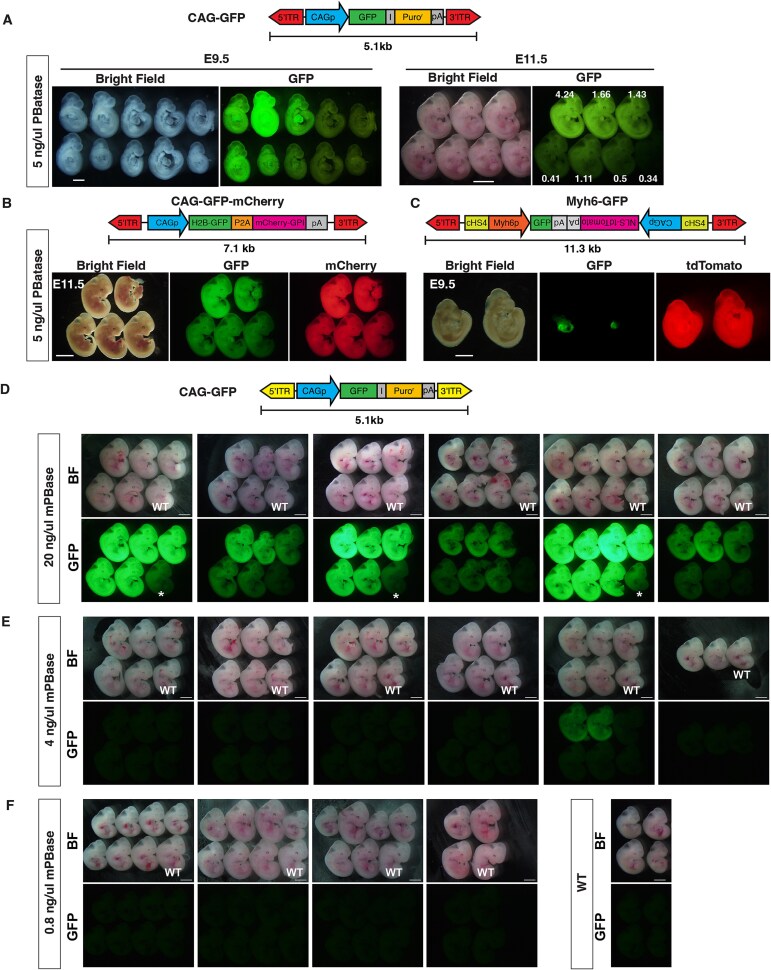
Generation of transgenic embryos with PBatase. (A) Fluorescence images of mouse embryos generated by *piggyBat*. The CAG-EGFP-IRES-Puro (CAG-GFP) donor DNA was introduced into fertilized mouse eggs with 5 ng/μl PBatase mRNA, and embryos at E9.5 (left) and E11.5 (right) were dissected. The transgene copy number annotations were also added to all E11.5 embryos of the right panel. Scale bar = 100 μm at E9.5 and 2 mm at E11.5. (B) Fluorescence image of mouse embryos with the CAG-GFP-mCherry transgene at E11.5. Above is shown the transgene construct. Note that GFP and mCherry were ubiquitously expressed. (C) Fluorescence image of Myh6-GFP transgenic embryos at E9.5. Above is shown the transgene construct. Note that tdTomato was ubiquitously expressed, and GFP was abundantly expressed in the heart. (D–F) Fluorescence images of mouse embryos generated by *piggyBac*. CAG-GFP donor DNA was introduced into fertilized mouse eggs together with mPBase mRNA at 20 ng/μl (D), 4 ng/μl (E), and 0.8 ng/μl (F). All fluorescence images in (D–F) were acquired under identical imaging conditions. The asterisk in (D) indicates WT embryos that appear faintly GFP-positive when placed adjacent to strongly fluorescent transgenic embryos. This may be due to reflection or scattering of GFP fluorescence by the surface or internal tissues of the WT embryos. A control image of WT embryos alone is shown in the right panel of (E).

Next, we attempted to obtain transgenic embryos at the post-implantation stage (Figure 3A). Forty-eight two-cell stage embryos were transferred to recipient mice, and 20 embryos were obtained at E9.5 (Table 2). Twelve embryos showed ubiquitous GFP expression (the representative 10 embryos are shown in Figure 3A, *left*). PCR genotyping indicated that 14 embryos were transgenic (Supplementary Figure 2A, Table 2), suggesting that the transgene in two of the transgenic embryos may be expressed at a very low level or silenced. At E11.5, 96 two-cell stage embryos were transferred to recipient mice and seven embryos were obtained. All seven embryos showed ubiquitous GFP expression (Figure 3A, right, Table 2). The intensity of GFP fluorescence appears to correlate with transgene copy number (Figure 3A, right).

**Table 2:** Summary of transgenic mice production experiments using PBase or PBatase system

	**Materials**						**Number**									
	**Transposase**	**mRNA conc. (ng/μl)**	**Donor plasmid DNA**				**Electroporated**	**Survived (% of electroporated)**	**Micro-injected**	**Survived (% of micro-injected)**	**Two-cell stage (% of survived)**	**Transplanted**	**Offspring (% of transplanted)**			**Transgenic (% of offspring)**
			**Construct**	**Plasmid size (kbp)**	**Insert size (5′ITR-to3′ITR, kbp)**	**Conc. (ng/μl)**							**E9.5**	**E11.5**	**Born**	
**Experiment**	mPBase	0.8	CAG-GFP	8.1	5.1	5	101	101 (100)	91	81 (89.0)	81 (100)	72	-	27 (37.5)	-	0^**^ (0)
		4	CAG-GFP	8.1	5.1	5	101	98 (97.0)	95	76 (80.0	76 (100)	72	-	26 (36.1)	-	3 (11.5)
		20	CAG-GFP	8.1	5.1	5	100	100 (100)	97	62 (63.9)	62 (100)	62	-	33 (53.2)	-	33 (100)
	PBatase	5	CAG-GFP	7.6	4.6	5	64	61 (95.3)	61	60 (98.4)	60 (100)	48	20 (41.7)			14 (70)
		5	CAG-GFP	7.6	4.6	5	31	30 (96.8)	28	26 (92.9)	24 (92.3)	24	-	7 (29.2)	-	7 (100)
		5	CAG-GFP	7.6	4.6	5	92	90 (97.8)	85	78 (91.8)	78 (100)	72	-	-	26 (36.1)	18^**^ (65)
		5	CAG-GFP-mCherry	9.8	7.1	7.7	316^***^	313 (99.1)	88	73 (83.0)	69 (94.5)	73^*^	-	10 (14.5)	-	10 (100)
		5	Myh6-GFP	14	11.3	12.3			194	128 (66.0)	102 (79.7)	128^*^ (88 for E9.5, 40 for E11.5)	16 (18.2)	13 (32.5)	-	29 (100)
**Negative control (no transposase)**	None	0	CAG-GFP	8.1	5.1	5	62	61 (98.4)	61	49 (80.3)	48 (98.0)	48	10 (20.8)	-	-	0^**^ (0)
**Negative control (no treatment)**	None	-	-	-	-	-	None	-	-	-	-	48	22 (45.8)	-	-	0^**^ (0)

^^*^^Including one-cell stage and two-cell stage embryos.

^^**^^Judged by only fluorescence microscope observation.

^^***^^Shared between CAG-GFP-mCherry and Myh6-GFP experiments.

Since transgenic embryos were obtained at E9.5 and E11.5 with high efficiency (70% at E9.5 and 100% at E11.5), we applied this strategy to deliver other transgenes with *piggyBat* (Figure 3B and C). After injection of a 7.1 kb *piggyBat* transposon ubiquitously expressing H2B-GFP and membrane-bound mCherry, 73 two-cell stage embryos were transferred into recipient mice. Ten embryos were dissected at E11.5, and 9 out of 10 (90%) ubiquitously expressed GFP and mCherry (Figure 3B). After injection of an 11.3 kb *piggyBat* transposon carrying tdTomato under a ubiquitous promoter and GFP under the heart-specific myosin heavy chain promoter, 88 two-cell stage embryos were transferred to recipient mice and 16 E9.5 embryos were obtained. Ten out of 16 embryos (62.5%) expressed tdTomato ubiquitously and GFP specifically in the heart (Figure 3C). Genotyping PCR indicated that all of these 16 embryos were transgenic (Supplementary Figure 2B, Table 2).

Previously, using *piggyBac*, we established a strategy to generate transgenic animals with nearly 100% efficiency [11]. The transgene copy numbers were 9.57 ± 2.17 at 100 ng/μl mPBase, 12.36 ± 1.75 at 500 ng/μl, and 7.41 ± 1.0 at 1000 ng/μl [11]. To investigate transposition activity at lower doses of mPBase (0.8, 4, and 20 ng/μl), electroporation, embryo transfer, and dissection experiments were performed at each concentration (Figure 3D–F). Fluorescence microscopy revealed that GFP-expressing transgenic animals were obtained at 4 and 20 ng/μl but not at 0.8 ng/μl (Figure 3D–F). Genotyping PCR showed Tg generation efficiency of 12% (3 Tg embryos out of 26) at 4 ng/μl and 100% (33 out of 33) at 20 ng/μl (Supplementary Figure 2C, Table 2).

### Estimation of transgene copy number in the mouse genome

**Figure 4:**
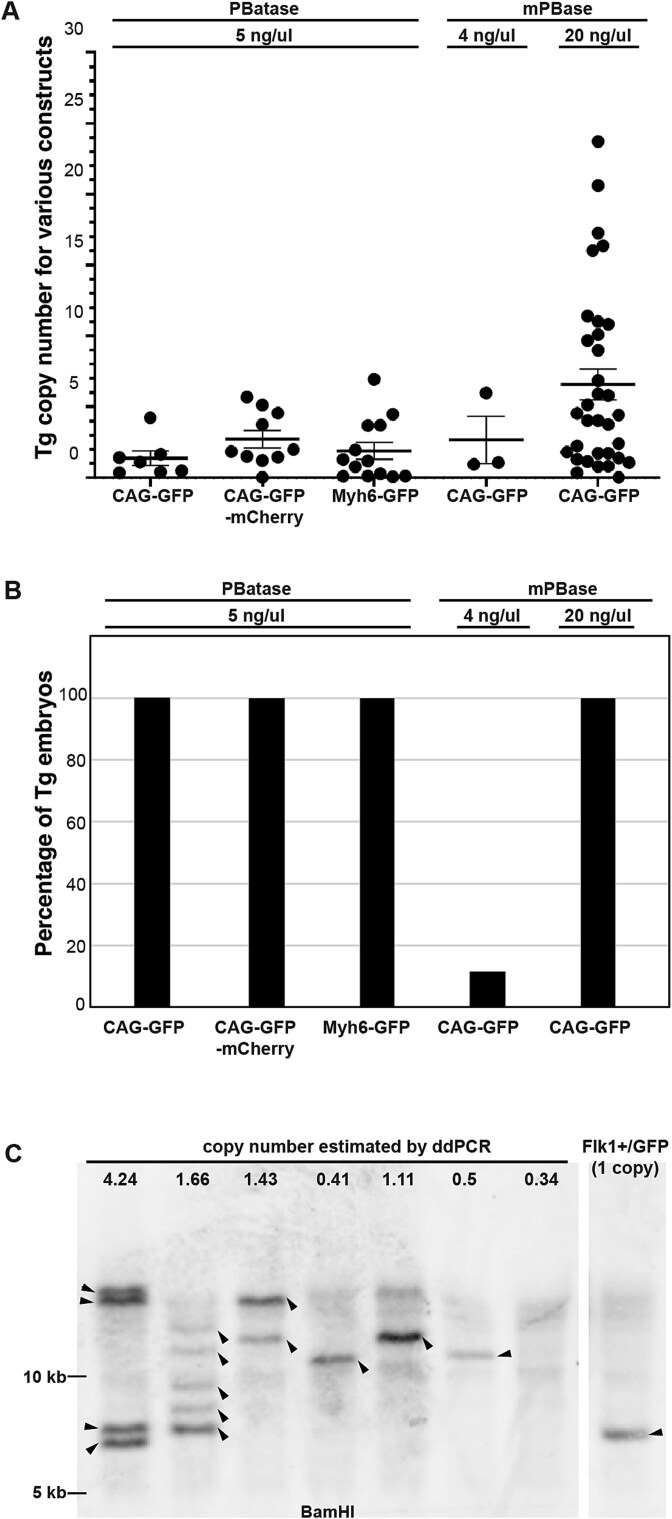
Analysis of transgene copy number in PBatase-generated embryos. (A) Transgene copy number in the E11.5 embryos generated by *piggyBat* and *piggyBac*. The copy numbers of the four transgene constructs shown in Figure 3 were analyzed by ddPCR. Data are represented as the mean ± SEM. (B) Percentage of Tg embryos generated by *piggyBat* and *piggyBac* shown in (A). (C) Southern blot analysis of DNA from the E11.5 transgenic embryos shown in Figure 3. The number shown at the top of the blot is the copy number measured by ddPCR analysis. Arrowheads show each transgene integrated in the genome by PBatase. *Flk1+/GFP* was used as a single-copy control.

Digital droplet PCR analysis showed that the PBatase-generated transgene copy number was 1.38 ± 0.51 for CAG-GFP, 2.72 ± 0.61 for CAG-GFP-mCherry and 1.90 ± 0.60 for Myh6-GFP, compared to an autosomal reference gene (Figure 4A). Low doses of mPBase resulted in transgene copy numbers of 2.66 ± 1.6 at 4 ng/μl and 5.9 ± 0.94 at 20 ng/μl. These results indicate that transgenic animals with low copy numbers (2.66 ± 1.6) can be generated by reducing the concentration of mPBase mRNA, although the efficiency is quite low (12%) (Figure 4B). In contrast, mPBase at 20 ng/μl achieves a high efficiency of 100% but results in a higher copy number (5.9 ± 0.94). Furthermore, when we compared the efficiency of transgenic animal generation using mPBase mRNA at 20 ng/μl and PBatase mRNA at 5 ng/μl, PBatase yielded transgenic embryos with significantly lower variability in transgene copy number, as determined by the Brown–Forsythe test [*F*(1, 38) = 4.60, *P* = 0.0385] (Figure 4A). Although mPBase at 20 ng/μl enabled high-efficiency transgenesis, it frequently resulted in animals with high transgene copy numbers (Figure 4A), which often necessitate euthanasia due to undesirable phenotypes. In contrast, PBatase efficiently generated transgenic animals with low copy numbers, reducing the need for animal exclusion and facilitating the establishment of stable transgenic lines.

Southern blot analysis of genomic DNA from individual embryos showed multiple bands, consistent with the ddPCR results. The total number of bands was greater than the transgene copy number estimated by ddPCR, indicating mosaicism of transgene integration in single embryos (Figure 4C). In addition, the overall intensity of the bands in the *piggyBat*-generated embryos varied within the same sample and many were lower than that of the GFP band in the Flk1+/GFP knock-in heterozygote [18] carrying a single copy of GFP (Figure 4C).

### Germline transmission from F0 mice

**Figure 5:**
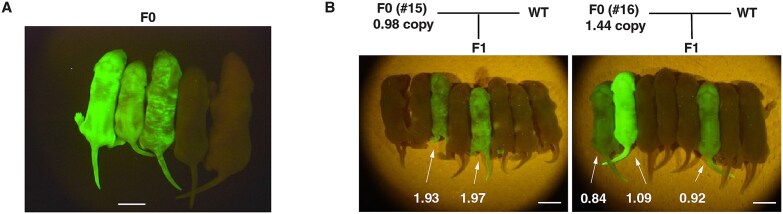
Germline transmission of the *piggyBat*-inserted transgene. (A) Fluorescence image of neonatal F0 generation transgenic mice. The four mice on the left side of the photo are F0 generation mice. The one on the right is a control wild-type mouse (ICR). (B) Fluorescence image of neonatal F1 generation transgenic mice. The number indicated for each transgenic mouse is the copy number determined by ddPCR analysis. Scale bars = 1 cm.

Transgenic animal generation using the *piggyBat* transposon is highly efficient, and most transgenic mice carry relatively few copies of the transgene. We therefore considered that the technique might be useful to generate independent transgenic lines. To test this idea, 72 two-cell stage embryos were transferred to recipient mice and 26 offspring were obtained. Eighteen out of 26 pups (69.2%) expressed GFP in the skin (Figure 5A). When two F0 mice were crossed with wild-type mice, two independent F1 mice were successfully obtained from each F0 mice (Figure 5B left and right). The transgene copy number was 1 or 2 (Figure 5B). Furthermore, GFP fluorescence was clearly observed in the skin of F1 pups (Figure 5B), indicating that the transgene was not only transmitted but also stably expressed in the germline-derived progeny. These results support the stable integration and expression of the transgene in the F1 generation.

## Discussion

We have shown that *piggyBat* exhibits detectable transposition activity in cultured mouse ESCs and early mouse embryos within a limited concentration range, whereas *piggyBac* demonstrates consistently higher activity over a broader range of doses. Notably, *piggyBat* activity peaked at moderate concentrations and declined at higher levels, likely due to overproduction inhibition. We have also shown that *piggyBat* can efficiently insert transgenes over 11 kb that produce tissue-specific expression of the exogenous transgene. We were able to derive transgenic founder lines that achieved germline transmission to the next generation, demonstrating that this strategy is a powerful tool for generating transgenic animals.

*piggyBac* and hyPBase, a hyperactive derivative of PBase [19], are widely used to generate transgenic animals, from experimental animals to livestock [20, 21]. However, the transgene copy number resulting from hyPBase is generally very high, making it difficult to achieve transgene segregation in the next generation and to produce a founder population carrying an identical exogenous gene. Our *piggyBat* and PBatase strategy, however, achieves both high efficiency and moderate transposition activity, making it useful to derive independent transgenic lines with a single transgene copy in fewer generations.

Overproduction inhibition is a regulatory mechanism in transposase-mediated transposition of transposon DNA, where excessive production of transposase reduces transposition efficiency and is commonly observed in bacterial transposable elements such as Tn5, Tn10, and the Mu transposon system [22]. Overproduction inhibition is also observed in both *piggyBac* and *Sleeping beauty* transposase [23], although there are conflicting reports [24]. Although Sutrave and coworkers reported that *piggyBat* has no apparent OPI in CAR19 expansion [13], we observed that *piggyBat* has significant OPI in our system. The discrepancy may be due to the difference in cell type used for the assay, and the optimal dose of *piggyBat* is very low.

In addition to confirming germline transmission, we observed that F1 animals maintained clear GFP fluorescence in the skin and carried only one or two copies of the transgene, indicating stable expression and inheritance in the next generation. Although additional studies in F2 or later generations would help validate long-term transgene stability, our findings support the utility of *piggyBat*-mediated transgenesis for establishing stable transgenic lines. Furthermore, the moderate activity and efficient single-copy integration demonstrated by *piggyBat* in mice suggest that this system may also hold promise for application in larger animal models, such as livestock. While additional optimization would likely be required to address species-specific embryonic handling, transposition efficiency, and regulatory challenges, *piggyBat* offers a potentially valuable platform for precise and efficient transgenesis beyond rodents.

Taken together, our study clearly indicates that *piggyBat* transposition is a useful strategy for generating transgenic animals.

## Supplementary Material

Supplementary_Figures_ioaf235

## Data Availability

Any additional data are available from the corresponding author upon reasonable request.
