# Supplementary material for: Efficient generation of single-copy transgenic mice using piggyBat transposase from the little brown bat Myotis lucifugus
Source: Biol Reprod. 2025 Sep 13;114(3):784–93. doi: 10.1093/biolre/ioaf235 (PMC13017672; doi:10.1093/biolre/ioaf235)
Supplement: Supplementary_Figures_ioaf235 [file supplementary_figures_ioaf235.pdf]

Figure Legends for Supplementary Figures

Supplementary Figure 1. Sequence structures for mPBase and PBatase

The ORF sequences of mPBase and PBatase shown in red were cloned into the pmRNA plasmid vector, which contains synthetic 5′ untranslated region (UTR) and mouse Hba-a1 gene 3′ UTR. Underline indicates T7 primer.

Supplementary Figure 2. PCR genotyping

A. Genotyping results for the embryos in Figure 3A right. 14 embryos were transgenic. B. All of 16 embryos in Figure 3C were transgenic. C. Genotyping PCR for the embryos in Figure 3D-F showed the efficiency of transgenic generation to be 12% (3 Tg embryos out of 26) at 4 ng/μl and 100% (33 out of 33) at 20 ng/μl.

>5'UTR-mPBase_CDS-3'UTR-pmRNA plasmid

```
TCGCGCGTTTCGGTGATGACGGTGAAAACCTCTGACACATGCAGCTCCCGGAGACGGTCACAGCTTGTCTGTAAGCGG
ATGCCGGGAGCAGACAAGCCCGTCAGGGCGCGTCAGCGGGTGTTGGCGGGTGTCGGGGCTGGCTTAACTATGCGGCAT
CAGAGCAGATTGTACTGAGAGTGCACCATATGCGGTGTGAAATACCGCACAGATGCGTAAGGAGAAAATACCGCATCA
GGCGCCATTCGCCATTCAGGCTGCGCAACTGTTGGGAAGGGCGATCGGTGCGGGCCTCTTCGCTATTACGCCAGCTGG
CGAAAGGGGGATGTGCTGCAAGGCGATTAAGTTGGGTAACGCCAGGGTTTTCCCAGTCACGACGTTGTAAAACGACGG
CCAGTGAATTCGAGCTCGGTACCTAATACGACTCACTATAGGGAAATAAGAGAGAAAAGAAGAGTAAGAAGAAATATA
AGAGCCACCATGGGTAGTTCTTTAGACGATGAGCATATCCTCTCTGCTCTTCTGCAAAGCGATGACGAGCTTGTTGGT
GAGGATTCTGACAGTGAAATATCAGATCACGTAAGTGAAGATGACGTCCAGAGCGATACAGAAGAAGCGTTTATAGAT
GAGGTACATGAAGTGCAGCCAACGTCAAGCGGTAGTGAAATATTAGACGAACAAAATGTTATTGAACAACCAGGTTCT
TCATTGGCTTCTAACAGAATCTTGACCTTGCCACAGAGGACTATTAGAGGTAAGAATAAACATTGTTGGTCAACTTCA
AAGTCCACGAGGCGTAGCCGAGTCTCTGCACTGAACATTGTCAGATCTCAAAGAGGTCCGACGCGTATGTGCCGCAAT
ATATATGACCCACTTTTATGCTTCAAACTATTTTTTACTGATGAGATAATTTCGGAAATTGTAAAATGGACAAATGCT
GAGATATCATTGAAACGTCGGGAATCTATGACAGGTGCTACATTTCGTGACACGAATGAAGATGAAATCTATGCTTTC
TTTGGTATTCTGGTAATGACAGCAGTGAGAAAAGATAACCACATGTCCACAGATGACCTCTTTGATCGATCTTTGTCA
ATGGTGTACGTCTCTGTAATGAGTCGTGATCGTTTTGATTTTTTGATACGATGTCTTAGAATGGATGACAAAAGTATA
CGGCCCACACTTCGAGAAAACGATGTATTTACTCCTGTTAGAAAAATATGGGATCTCTTTATCCATCAGTGCATACAA
AATTACACTCCAGGGGCTCATTTGACCATAGATGAACAGTTACTTGGTTTTAGAGGACGGTGTCCGTTTAGGATGTAT
ATCCCAAACAAGCCAAGTAAGTATGGAATAAAAATCCTCATGATGTGTGACAGTGGTACGAAGTATATGATAAATGGA
ATGCCTTATTTGGGAAGAGGAACACAGACCAACGGAGTACCACTCGGTGAATACTACGTGAAGGAGTTATCAAAGCCT
GTGCACGGTAGTTGTCGTAATATTACGTGTGACAATTGGTTCACCTCAATCCCTTTGGCAAAAAACTTACTACAAGAA
CCGTATAAGTTAACCATTGTGGGAACCGTGCGATCAAACAAACGCGAGATACCGGAAGTACTGAAAAACAGTCGCTCC
AGGCCAGTGGGAACATCGATGTTTTGTTTTGACGGACCCCTTACTCTCGTCTCATATAAACCGAAGCCAGCTAAGATG
GTATACTTATTATCATCTTGTGATGAGGATGCTTCTATCAACGAAAGTACCGGTAAACCGCAAATGGTTATGTATTAT
AATCAAACTAAAGGCGGAGTGGACACGCTAGACCAAATGTGTTCTGTGATGACCTGCAGTAGGAAGACGAATAGGTGG
CCTATGGCATTATTGTACGGAATGATAAACATTGCCTGCATAAATTCTTTTATTATATACAGCCATAATGTCAGTAGC
AAGGGAGAAAAGGTTCAAAGTCGCAAAAAATTTATGAGAAACCTTTACATGAGCCTGACGTCATCGTTTATGCGTAAG
CGTTTAGAAGCTCCTACTTTGAAGAGATATTTGCGCGATAATATCTCTAATATTTTGCCAAATGAAGTGCCTGGTACA
TCAGATGACAGTACTGAAGAGCCAGTAATGAAAAAACGTACTTACTGTACTTACTGCCCCTCTAAAATAAGGCGAAAG
GCAAATGCATCGTGCAAAAAATGCAAAAAAGTTATTTGTCGAGAGCATAATATTGATATGTGCCAAAGTTGTTTCTGA
GCGGCCGCTTAATTAAGCTGCCTTCTGCGGGGCTTGCCTTCTGGCCATGCCCTTCTTCTCTCCCTTGCACCTGTACCT
CTTGGTCTTTGAATAAAGCCTGAGTAGGAAGTCTAGAGTTTAAACATTTAAATCTGCAGAGGCCTGCATGCAAGCTTG
GCGTAATCATGGTCATAGCTGTTTCCTGTGTGAAATTGTTATCCGCTCACAATTCCACACAACATACGAGCCGGAAGC
ATAAAGTGTAAAGCCTGGGGTGCCTAATGAGTGAGCTAACTCACATTAATTGCGTTGCGCTCACTGCCCGCTTTCCAG
TCGGGAAACCTGTCGTGCCAGCTGCATTAATGAATCGGCCAACGCGCGGGGAGAGGCGGTTTGCGTATTGGGCGCTCT
TCCGCTTCCTCGCTCACTGACTCGCTGCGCTCGGTCGTTCGGCTGCGGCGAGCGGTATCAGCTCACTCAAAGGCGGTA
ATACGGTTATCCACAGAATCAGGGGATAACGCAGGAAAGAACATGTGAGCAAAAGGCCAGCAAAAGGCCAGGAACCGT
AAAAAGGCCGCGTTGCTGGCGTTTTTCCATAGGCTCCGCCCCCCTGACGAGCATCACAAAAATCGACGCTCAAGTCAG
AGGTGGCGAAACCCGACAGGACTATAAAGATACCAGGCGTTTCCCCCTGGAAGCTCCCTCGTGCGCTCTCCTGTTCCG
ACCCTGCCGCTTACCGGATACCTGTCCGCCTTTCTCCCTTCGGGAAGCGTGGCGCTTTCTCATAGCTCACGCTGTAGG
TATCTCAGTTCGGTGTAGGTCGTTCGCTCCAAGCTGGGCTGTGTGCACGAACCCCCCGTTCAGCCCGACCGCTGCGCC
TTATCCGGTAACTATCGTCTTGAGTCCAACCCGGTAAGACACGACTTATCGCCACTGGCAGCAGCCACTGGTAACAGG
ATTAGCAGAGCGAGGTATGTAGGCGGTGCTACAGAGTTCTTGAAGTGGTGGCCTAACTACGGCTACACTAGAAGAACA
GTATTTGGTATCTGCGCTCTGCTGAAGCCAGTTACCTTCGGAAAAAGAGTTGGTAGCTCTTGATCCGGCAAACAAACC
ACCGCTGGTAGCGGTGGTTTTTTTGTTTGCAAGCAGCAGATTACGCGCAGAAAAAAAGGATCTCAAGAAGATCCTTTG
ATCTTTTCTACGGGGTCTGACGCTCAGTGGAACGAAAACTCACGTTAAGGGATTTTGGTCATGAGATTATCAAAAAGG
ATCTTCACCTAGATCCTTTTAAATTAAAAATGAAGTTTTAAATCAATCTAAAGTATATATGAGTAAACTTGGTCTGAC
AGTTACCAATGCTTAATCAGTGAGGCACCTATCTCAGCGATCTGTCTATTTCGTTCATCCATAGTTGCCTGACTCCCC
GTCGTGTAGATAACTACGATACGGGAGGGCTTACCATCTGGCCCCAGTGCTGCAATGATACCGCGAGACCCACGCTCA
CCGGCTCCAGATTTATCAGCAATAAACCAGCCAGCCGGAAGGGCCGAGCGCAGAAGTGGTCCTGCAACTTTATCCGCC
TCCATCCAGTCTATTAATTGTTGCCGGGAAGCTAGAGTAAGTAGTTCGCCAGTTAATAGTTTGCGCAACGTTGTTGCC
ATTGCTACAGGCATCGTGGTGTCACGCTCGTCGTTTGGTATGGCTTCATTCAGCTCCGGTTCCCAACGATCAAGGCGA
GTTACATGATCCCCCATGTTGTGCAAAAAAGCGGTTAGCTCCTTCGGTCCTCCGATCGTTGTCAGAAGTAAGTTGGCC
GCAGTGTTATCACTCATGGTTATGGCAGCACTGCATAATTCTCTTACTGTCATGCCATCCGTAAGATGCTTTTCTGTG
ACTGGTGAGTACTCAACCAAGTCATTCTGAGAATAGTGTATGCGGCGACCGAGTTGCTCTTGCCCGGCGTCAATACGG
GATAATACCGCGCCACATAGCAGAACTTTAAAAGTGCTCATCATTGGAAAACGTTCTTCGGGGCGAAAACTCTCAAGG
ATCTTACCGCTGTTGAGATCCAGTTCGATGTAACCCACTCGTGCACCCAACTGATCTTCAGCATCTTTTACTTTCACC
AGCGTTTCTGGGTGAGCAAAAACAGGAAGGCAAAATGCCGCAAAAAAGGGAATAAGGGCGACACGGAAATGTTGAATA
CTCATACTCTTCCTTTTTCAATATTATTGAAGCATTTATCAGGGTTATTGTCTCATGAGCGGATACATATTTGAATGT
ATTTAGAAAAATAAACAAATAGGGGTTCCGCGCACATTTCCCCGAAAAGTGCCACCTGACGTCTAAGAAACCATTATT
ATCATGACATTAACCTATAAAAATAGGCGTATCACGAGGCCCTTTCGTC
```

Okamura et al., Supplementary Fig. S1

>5’UTR-PBatase_CDS-3’UTR- pmRNA plasmid

TCGCGCGTTTCGGTGATGACGGTGAAAACCTCTGACACATGCAGCTCCCGGAGACGGTCACAGCTTGTCTGTAAGCGG
ATGCCGGGAGCAGACAAGCCCGTCAGGGCGCGTCAGCGGGTGTTGGCGGGTGTCGGGGCTGGCTTAACTATGCGGCAT
CAGAGCAGATTGTACTGAGAGTGCACCATATGCGGTGTGAAATACCGCACAGATGCGTAAGGAGAAAATACCGCATCA
GGCGCCATTCGCCATTCAGGCTGCGCAACTGTTGGGAAGGGCGATCGGTGCGGGCCTCTTCGCTATTACGCCAGCTGG
CGAAAGGGGGATGTGCTGCAAGGCGATTAAGTTGGGTAACGCCAGGGTTTTCCCAGTCACGACGTTGTAAAACGACGG
CCAGTGAATTCGAGCTCGGTACCTAATACGACTCACTATAGGGAAATAAGAGAGAAAAGAAGAGTAAGAAGAAATATA
AGAGCCACCATGGCGCAACACTCAGATTACTCCGACGATGAATTTTGTGCTGACAAACTGTCCAATTATTCATGCGAT
AGCGACCTCGAAAACGCTTCCACGTCTGATGAAGATAGCAGCGATGATGAAGTAATGGTGAGGCCTCGCACCCTCCGC
CGTCGCCGCATCAGCTCTTCGAGCTCTGATTCTGAATCCGATATTGAGGGTGGCCGCGAGGAGTGGTCCCACGTAGAC
AATCCGCCGGTGCTGGAGGACTTCCTAGGCCACCAAGGTCTGAACACTGACGCAGTAATCAACAATATCGAAGATGCA
GTTAAACTGTTTATCGGTGACGATTTCTTCGAGTTTCTGGTGGAGGAATCTAACCGGTACTATAACCAGAATCGTAAT
AACTTCAAGCTCTCTAAAAAGTCTCTGAAGTGGAAGGACATCACCCCTCAGGAGATGAAAAAGTTCCTCGGTCTGATC
GTTCTGATGGGCCAAGTTCGCAAGGATCGTCGTGACGACTATTGGACTACCGAACCGTGGACGGAAACTCCATACTTT
GGCAAGACCATGACTCGTGACCGTTTCCGTCAGATCTGGAAGGCCTGGCACTTCAATAACAACGCTGACATTGTCAAC
GAGTCTGATCGTCTGTGTAAGGTTCGCCCTGTGCTGGATTACTTCGTTCCAAAATTCATTAACATTTACAAACCACAT
CAGCAGCTGTCCCTGGATGAGGGCATCGTGCCGTGGCGCGGCCGCCTGTTCTTCCGTGTCTATAATGCTGGCAAGATT
GTGAAGTACGGTATCCTGGTTCGCCTGCTGTGCGAAAGCGACACTGGCTACATCTGTAACATGGAGATCTACTGCGGC
GAGGGCAAACGTCTCCTCGAAACTATCCAGACCGTCGTGTCTCCATACACGGATTCCTGGTATCATATTTACATGGAT
AACTATTATAACAGCGTGGCTAACTGTGAAGCTCTGATGAAAAATAAGTTCCGTATTTGCGGTACTATCCGTAAGAAT
CGTGGAATTCCGAAAGATTTCCAGACCATCTCCCTGAAAAAGGGTGAAACTAAGTTCATTCGCAAAAACGACATCCTC
CTGCAAGTCTGGCAGTCTAAAAAGCCTGTATATCTGATCTCATCTATTCACAGCGCTGAAATGGAAGAATCTCAGAAC
ATTGATCGCACCTCCAAGAAAAAGATCGTCAAACCGAATGCATTGATTGATTACAACAAGCACATGAAGGGCGTTGAT
CGTGCTGACCAGTACCTGTCTTATTACTCTATCCTGCGCCGTACTGTGAAGTGGACTAAACGTCTCGCTATGTACATG
ATTAATTGTGCGCTGTTCAATTCTTACGCTGTGTATAAAAGCGTGCGTCAGCGCAAAATGGGCTTTAAAATGTTCCTG
AAGCAGACGGCTATTCACTGGCTGACCGACGATATTCCGGAAGATATGGACATTGTCCCGGATCTCCAGCCGGTACCG
AGCACCAGCGGTATGCGTGCTAAACCTCCGACTAGTGATCCGCCTTGCCGTCTGTCTATGGATATGCGTAAGCATACC
CTGCAGGCAATTGTGGGCTCTGGCAAAAAGAAAAATATCCTGCGTCGTTGCCGCGTATGCTCTGTACACAAACTGCGT
TCTGAGACTCGTTATATGTGTAAATTTTGCAATATTCCACTCCACAAGGGTGCGTGCTTCGAGAAGTACCATACGCTG
AAGAACTATTCTAGATGAGCGGCCGCTTAATTAAGCTGCCTTCTGCGGGGCTTGCCTTCTGGCCATGCCCTTCTTCTC
TCCCTTGCACCTGTACCTCTTGGTCTTTGAATAAAGCCTGAGTAGGAAGTCTAGAGTTTAAACATTTAAATCTGCAGA
GGCCTGCATGCAAGCTTGGCGTAATCATGGTCATAGCTGTTTCCTGTGTGAAATTGTTATCCGCTCACAATTCCACAC
AACATACGAGCCGGAAGCATAAAGTGTAAAGCCTGGGGTGCCTAATGAGTGAGCTAACTCACATTAATTGCGTTGCGC
TCACTGCCCGCTTTCCAGTCGGGAAACCTGTCGTGCCAGCTGCATTAATGAATCGGCCAACGCGCGGGGAGAGGCGGT
TTGCGTATTGGGCGCTCTTCCGCTTCCTCGCTCACTGACTCGCTGCGCTCGGTCGTTCGGCTGCGGCGAGCGGTATCA
GCTCACTCAAAGGCGGTAATACGGTTATCCACAGAATCAGGGGATAACGCAGGAAAGAACATGTGAGCAAAAGGCCAG
CAAAAGGCCAGGAACCGTAAAAAGGCCGCGTTGCTGGCGTTTTTCCATAGGCTCCGCCCCCCTGACGAGCATCACAAA
AATCGACGCTCAAGTCAGAGGTGGCGAAACCCGACAGGACTATAAAGATACCAGGCGTTTCCCCCTGGAAGCTCCCTC
GTGCGCTCTCCTGTTCCGACCCTGCCGCTTACCGGATACCTGTCCGCCTTTCTCCCTTCGGGAAGCGTGGCGCTTTCT
CATAGCTCACGCTGTAGGTATCTCAGTTCGGTGTAGGTCGTTCGCTCCAAGCTGGGCTGTGTGCACGAACCCCCCGTT
CAGCCCGACCGCTGCGCCTTATCCGGTAACTATCGTCTTGAGTCCAACCCGGTAAGACACGACTTATCGCCACTGGCA
GCAGCCACTGGTAACAGGATTAGCAGAGCGAGGTATGTAGGCGGTGCTACAGAGTTCTTGAAGTGGTGGCCTAACTAC
GGCTACACTAGAAGAACAGTATTTGGTATCTGCGCTCTGCTGAAGCCAGTTACCTTCGGAAAAAGAGTTGGTAGCTCT
TGATCCGGCAAACAAACCACCGCTGGTAGCGGTGGTTTTTTTGTTTGCAAGCAGCAGATTACGCGCAGAAAAAAAGGA
TCTCAAGAAGATCCTTTGATCTTTTCTACGGGGTCTGACGCTCAGTGGAACGAAAACTCACGTTAAGGGATTTTGGTC
ATGAGATTATCAAAAAGGATCTTCACCTAGATCCTTTTAAATTAAAAATGAAGTTTTAAATCAATCTAAAGTATATAT
GAGTAAACTTGGTCTGACAGTTACCAATGCTTAATCAGTGAGGCACCTATCTCAGCGATCTGTCTATTTCGTTCATCC
ATAGTTGCCTGACTCCCCGTCGTGTAGATAACTACGATACGGGAGGGCTTACCATCTGGCCCCAGTGCTGCAATGATA
CCGCGAGACCCACGCTCACCGGCTCCAGATTTATCAGCAATAAACCAGCCAGCCGGAAGGGCCGAGCGCAGAAGTGGT
CCTGCAACTTTATCCGCCTCCATCCAGTCTATTAATTGTTGCCGGGAAGCTAGAGTAAGTAGTTCGCCAGTTAATAGT
TTGCGCAACGTTGTTGCCATTGCTACAGGCATCGTGGTGTCACGCTCGTCGTTTGGTATGGCTTCATTCAGCTCCGGT
TCCCAACGATCAAGGCGAGTTACATGATCCCCCATGTTGTGCAAAAAAGCGGTTAGCTCCTTCGGTCCTCCGATCGTT
GTCAGAAGTAAGTTGGCCGCAGTGTTATCACTCATGGTTATGGCAGCACTGCATAATTCTCTTACTGTCATGCCATCC
GTAAGATGCTTTTCTGTGACTGGTGAGTACTCAACCAAGTCATTCTGAGAATAGTGTATGCGGCGACCGAGTTGCTCT
TGCCCGGCGTCAATACGGGATAATACCGCGCCACATAGCAGAACTTTAAAAGTGCTCATCATTGGAAAACGTTCTTCG
GGGCGAAAACTCTCAAGGATCTTACCGCTGTTGAGATCCAGTTCGATGTAACCCACTCGTGCACCCAACTGATCTTCA
GCATCTTTTACTTTCACCAGCGTTTCTGGGTGAGCAAAAACAGGAAGGCAAAATGCCGCAAAAAAGGGAATAAGGGCG
ACACGGAAATGTTGAATACTCATACTCTTCCTTTTTCAATATTATTGAAGCATTTATCAGGGTTATTGTCTCATGAGC
GGATACATATTTGAATGTATTTAGAAAAATAAACAAATAGGGGTTCCGCGCACATTTCCCCGAAAAGTGCCACCTGAC
GTCTAAGAAACCATTATTATCATGACATTAACCTATAAAAATAGGCGTATCACGAGGCCCTTTCGTC

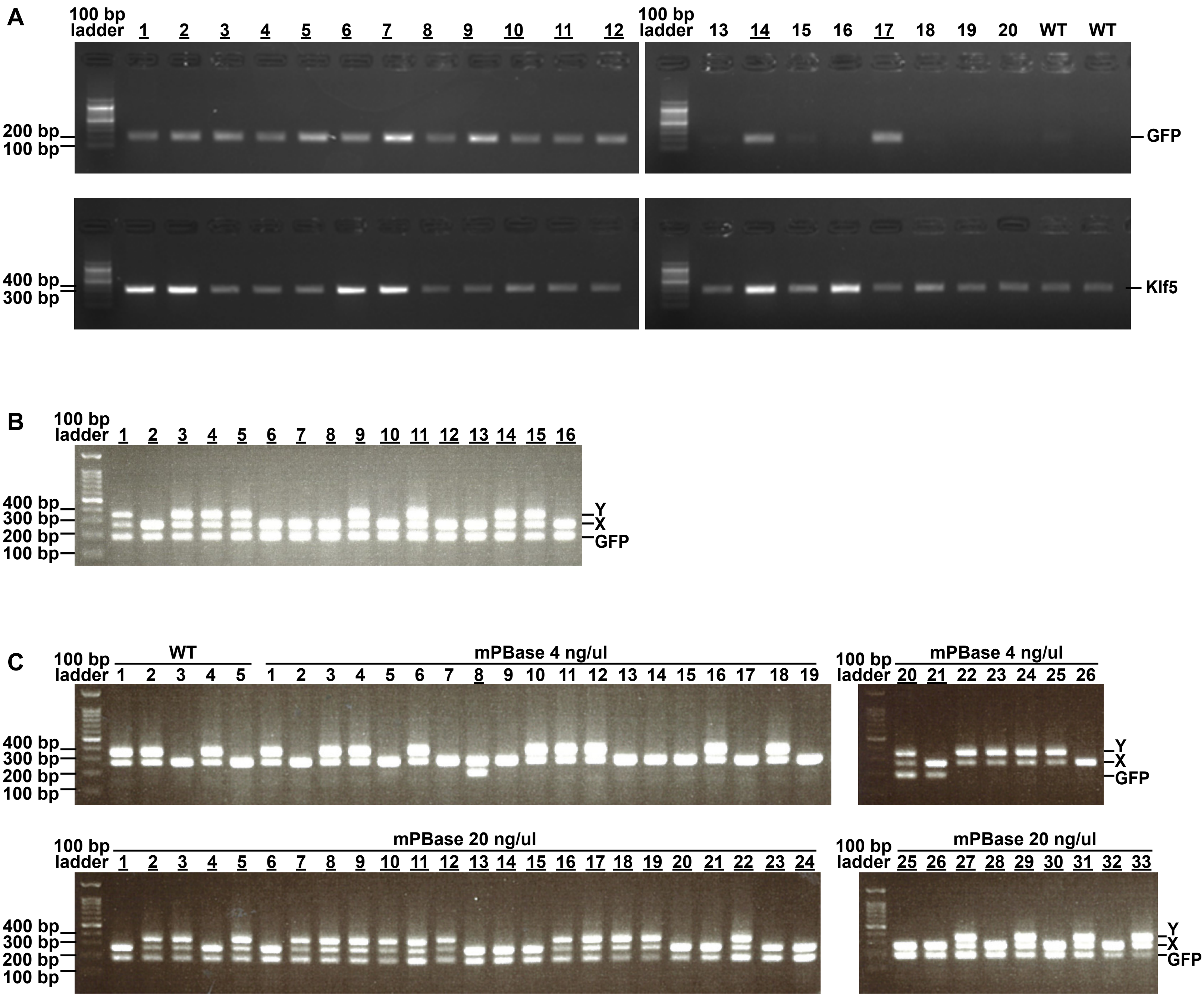

Okamura et al., Supplementary Fig. S2
